# miR-33 inhibition as a novel therapeutic approach for treating muscular dystrophy

**DOI:** 10.1038/s44321-025-00271-x

**Published:** 2025-07-23

**Authors:** Michael A Lopez, Matthew S Alexander

**Affiliations:** 1 Department of Pediatrics, Division of Neurology at the University of Alabama at Birmingham and Children’s of Alabama, Birmingham, AL 35294 USA; 2 UAB Center for Exercise Medicine at the University of Alabama at Birmingham, Birmingham, AL 35294 USA; 3 UAB Civitan International Research Center (CIRC), at the University of Alabama at Birmingham, Birmingham, AL 35233 USA; 4 UAB Center for Neurodegeneration and Experimental Therapeutics (CNET), Birmingham, AL 35294 USA

**Keywords:** Musculoskeletal System, RNA Biology

## Abstract

M. Alexander and M. Lopez discuss targeting miR-33a/b as a potential therapeutic strategy for muscular dystrophy as reported by K. Ono and colleagues, in this issue of *EMBO Mol Med*.

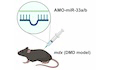

## Teaser

Duchenne muscular dystrophy (DMD) is a devastating disorder caused by pathogenic variants in the dystrophin gene resulting in the absence of a functional dystrophin protein. While the primary cause of DMD is well-documented, the impact of disrupted secondary signaling pathways in dystrophic muscles and organs is still being unraveled. MicroRNAs are small, non-coding RNAs known to regulate key signaling pathways in growth, regeneration, and disease. In this issue of EMBO Molecular Medicine, Sowa and colleagues report the inhibition of a key microRNA (miR-33a/b) that when inhibited can improve muscle regeneration and mitigate dystrophic symptoms in a DMD (*mdx*) mouse model. *miR-33a/b* knockout mice showed enhanced muscle regeneration response to cardiotoxin injury and attenuated muscle fibrosis on the *mdx* background. miR-33a/b muscle expression blocked muscle satellite cell proliferation impaired muscle regeneration while increasing by targeting *Cdk6*, *Fst*, and *Abca1* transcripts. Injection of anti-microRNA oligonucleotides (AMOs) targeting miR-33a/b improved muscle regeneration, histopathologies, and dystrophic regeneration signaling pathways. These studies demonstrate the impact of key secondary signaling pathways that may serve as new therapeutic avenues for the treatment of DMD symptoms.

Duchenne muscular dystrophy is caused by pathogenic variants (deletions, duplications, point variants, and other more rare variants) in the dystrophin (abbreviated *DMD*) gene that result in the loss of dystrophin protein. DMD patients suffer from a progressive muscle weakness leading towards the loss of ambulation by their teenage years, cardiopulmonary weakness, and eventual patient death by the third decade of life. The loss of a functional dystrophin protein has profound systemic effects on the skeletal muscles, heart, lungs, and other dystrophin-expressing tissues. The loss of dystrophin protein has been shown to result in a significant dysregulation of secondary signaling pathways that can also impact DMD disease progression and can serve as therapeutic targets for the disease. These non-coding RNAs (sometimes referred to dystromiRs) are microRNAs that often function to normally regulate skeletal muscle growth, regeneration, and overall development (Zaharieva et al, 2013). While many of these microRNAs are skeletal muscle-enriched (myomiRs) and are dysregulated in neuromuscular disorders (NMDs) such as DMD, many other microRNAs regulating fibrosis, inflammation, and even the dystrophin mRNA itself will affect DMD disease progression (Fiorillo et al, 2015; Kiełbowski et al, 2024). Several of these myomiRs, such as *miR-1, -133a/b, -206, -208a, -486, -489*, and *-499* all show the ability to modulate dystrophic pathologies by regulating target mRNA expression, thereby modulating DMD myofiber and muscle satellite cell (MuSC) function (Koopmans et al, 2023; Meng et al, 2022; Oikawa and Akimoto, 2023).

MicroRNA-33a and microRNA-33b are encoded within the mammalian *SREBF1* and *SRBF2* genes, respectively, and both SREBF1 and SRBF2 are essential for the export intracellular cholesterol to apolipoprotein A-I, leading to high-density lipoprotein cholesterol (HDL-C) formation within a cell (Price et al, 2017; Rayner et al, 2010).

**Figure 1:**
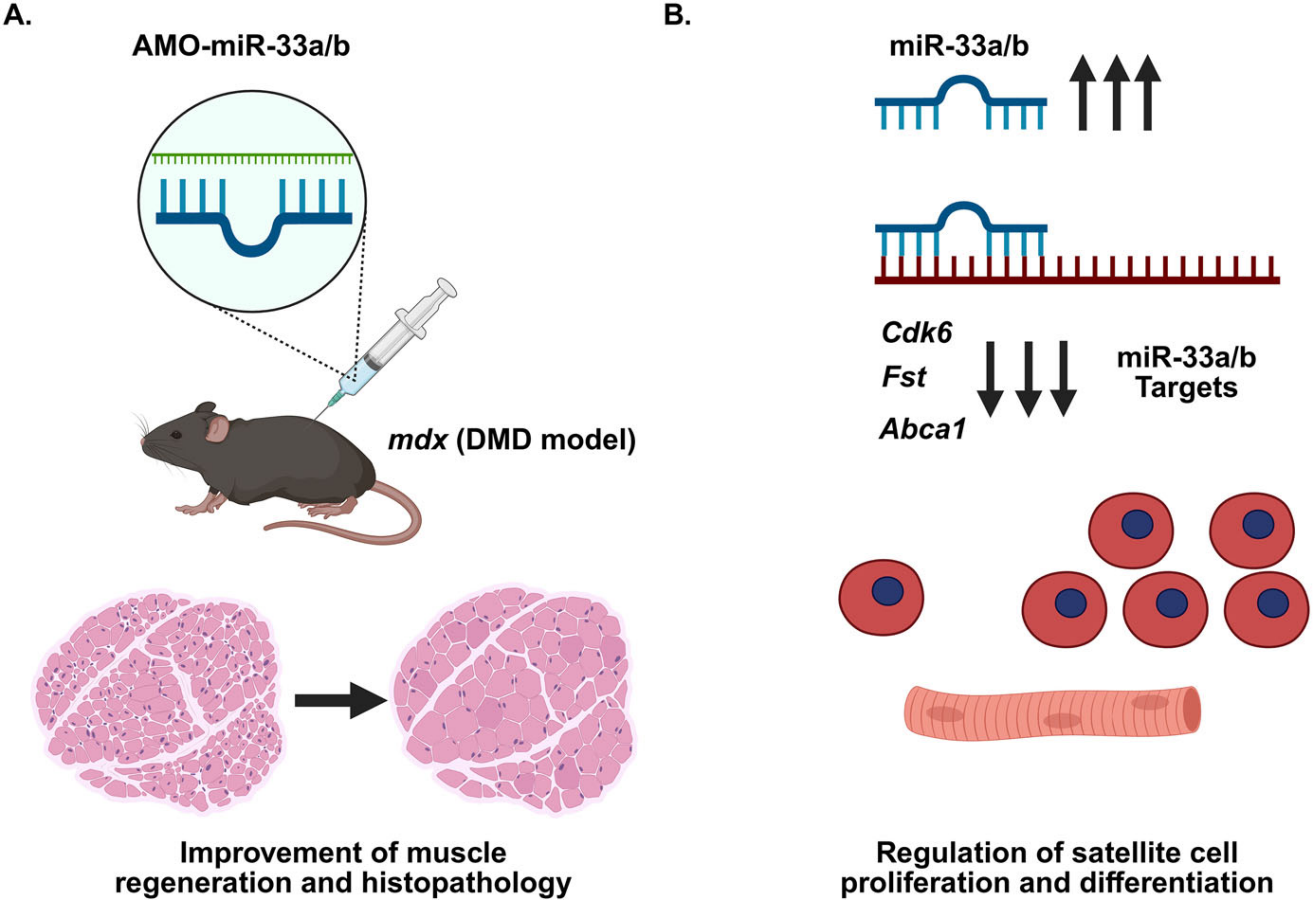
miR-33 inhibition as a strategy to improve muscular dystrophy symptoms. (**A**) Schematic showing that direct TA muscle injection using the AMO-miR-33a or AMO-miR-33b demonstrated improved skeletal muscle pathologies in the *mdx* (DMD) mice. Improvement in overall muscle histopathology and disease symptoms was observed 4 weeks post injection of the miR-33a/b inhibitors. (**B**) miR-33a/b overexpression inhibits mRNA transcripts (*Cdk6*, *Fst*, and *Abca1*) that regulate key cell cycle, quiescence and proliferation, and muscle repair/regeneration transcripts.

The authors of the current study (Sowa et al, 2025) previously generated *miR-33a/b* knockout mice and identified several key concepts with regard to miR-33 functional roles in muscle and dystrophin-deficiency (Fig. 1). miR-33a has similar expression levels to its host gene, *Srebf2*, in the liver and skeletal muscles (both slow and fast-twitch), and the authors first validated its expression and also demonstrated a concordant upregulation during C2C12 myogenic differentiation. Interestingly, while the *miR-33a* knockout (KO) mice had a minimal phenotype, the authors showed that administration of a cardiotoxin (CTX)-induced tibialis anterior (TA) injury, the *miR-33a* KO mice showed less signs of muscle damage than control mice one week post injury. The authors delved further into characterizing miR-33a function by generating *miR-33a/mdx* (*Dystrophin* exon 23 mutation) double mutant model. The *miR-33a/mdx* double knockout (dKO) mice displayed larger myofibers and reduced fibrosis, with reduced expression of muscle creatine kinase (CK), an elevated serum biomarker for DMD, compared to *mdx* control mice. Furthermore, the authors probed into the regulation of muscle satellite cells (SCs), which were more abundant in the *miR-33a/mdx* dKO mice with an accompanying increased myogenic markers (MyoD1, MyoG, and Pax7). The authors also evaluated a previously generated humanized *miR-33b*-KI mice, in which the human *miR-33b* sequence was inserted within the same intron of *Srebf1* (Horie et al, 2014). The *miR-33b*-KI/*mdx* double mutant mice showed reduced levels of muscle satellite cells, reduced Utrophin levels, and worsen muscle pathologies compared to *mdx* mice. The authors then used a candidate-based approach and identified 3 miR-33 target mRNAs: *Abca1*, *Cdk6*, and *Fst*. Each of these miR-33 target mRNAs was then evaluated using adeno-associated viral (AAV) vector shRNAi knockdown and demonstrated to exacerbate muscle pathology and regenerative capacity. Direct TA muscle injection using the amido-bridged nucleic acid (AmNA)-based artificial nucleotides (AMOs) targeting either miR-33a or miR-33b resulted in an upregulation of the miR-33 target RNAs, with a greater effect on expression levels in the miR-33b AMO-treated *miR-33b*-KI/*mdx* mice. The beneficial effects observed in the AmNA-miR-33-treated muscles led the authors to pursue transcriptomic analysis, which demonstrated key muscle cell cycle regulation, satellite cell activation/proliferation, muscle regeneration, and myogenic differentiation pathways. These findings supported the authors’ postulate that miR-33 inhibition could benefit muscle and in particular muscular dystrophy for which many of the symptoms were reduced overall.

Several important questions remain as to how to most effectively advance miR-33 inhibition strategies towards improving patient health in a viable drug-delivery strategy and platform. In addition, similar NMDs such as Becker muscular dystrophy (BMD), sarcoglycanopathies, and limb girdle muscular dystrophies (LGMDs) could potentially also benefit from miR-33 inhibition to improve muscle regeneration and pathologies. Sarcopenia and cachexia that are caused by aging or disease could also benefit from suppressing a common miRNA that regulates MuSC proliferation and muscle growth. Suppression of miR-33 could lead to improved or faster muscle regeneration in normal healthy muscle as well; however, it is important to note that the secondary targets of miR-33a/b in other non-muscle tissues may also be impacted unless tissue-specific knockdown can be achieved. The impact of miR-33 inhibition on improving cholesterol profiles in AMO-injected mice is also of interest as cholesterol metabolism has been implicated as a potential target for DMD (Amor et al, 2021). However, direct blockade of cholesterol using commonly prescribed statins has had mixed effects at best when evaluating dystrophic phenotypes in mdx mice and may not be effective when used in combination with currently approved micro-dystrophin gene therapy (Bourg et al, 2022).

In addition, microRNAs as therapeutic targets have had mixed results overall with regards to human clinical trial efficacy (Seyhan, 2024). Question with regards to the ability of a microRNA to achieve persistent expression in the target tissue(s) of interest, miRNA inhibitor chemistry and stability properties, and potential undesired off-target effects in other tissues remain. miR-33a and miR-33b likely have unique transcriptional regulation at the site of their host gene promoters that may impact their sustained expression in the context of disease and muscle regeneration. Defining the transcriptional and post-transcriptional regulation of miR-33a/b may lead to additional clues with regards to tissue-specificity and non-muscle target genes. Expanded contextual analysis of the miR-33a/b knockout and knock-in mice may elucidate what tissue symptoms and cell signaling pathways may also be druggable or serve as biomarkers for muscle and non-muscle diseases.
